# Green Synthesis of Zinc Oxide Nanoparticles Using *Viscum album* Extracts: Unveiling Bioactive Compounds, Antibacterial Potential, and Antioxidant Activities

**DOI:** 10.3390/plants12112130

**Published:** 2023-05-27

**Authors:** Waheeda Mushtaq, Muhammad Ishtiaq, Mehwish Maqbool, Muhammad Waqas Mazhar, Ryan Casini, Ahmed M. Abd-ElGawad, Hosam O. Elansary

**Affiliations:** 1 Department of Botany, Mirpur University of Science & Technology (MUST), Mirpur 10250, Pakistan; 2 School of Public Health, University of California, Berkeley, 2121 Berkeley Way, Berkeley, CA 94704, USA; 3 Department of Plant Production, College of Food & Agriculture Sciences, King Saud University, P.O. Box 2460, Riyadh 11451, Saudi Arabia

**Keywords:** green synthesis, zinc oxide nanoparticles, *Viscum album*, antimicrobial activities, phytomedicines

## Abstract

The current study explored the antioxidant and antibacterial capabilities of zinc oxide nanoparticles (ZnONPs) synthetized using methanolic leaf extracts of the medicinal herb *Viscum album*. Through TEM investigation and UV–Vis analysis, which peaked at 406 nm, the synthesis of ZnONPs was verified. TEM analyses showed that the synthesized ZnONPs had a size distribution with an average of 13.5 nm and a quasi-spherical shape. Forty-four phytoconstituents were found in the methanolic leaf extracts of *V. album*. Additionally, a comparison of the antibacterial effectiveness and antioxidant capacity of aqueous and methanolic extracts of wild-grown *V. album* phytomedicine and green-manufactured ZnONPs was conducted. The green-generated ZnONPs were examined against *Escherichia coli*, *Staphylococcus aureus*, and *Pseudomonas aeruginosa* and shown to have superior antibacterial activity by 22%, 66%, and 44%, respectively, as compared to wild herbal medicinal extracts. Since the ZnONPs’ aqueous extracts had higher concentrations of DNA gyrase-B inhibitory components, they were shown to be more effective in limiting bacterial growth. In contrast to the percentages of 49% and 57% for a wild plant extract, the aqueous- and methanolic-extract-mediated green ZnONPs, with a 100 g/mL concentration, showed 94% and 98% scavenging capacity for DPPH free radicals, respectively. However, methanolic extracts were more effective than aqueous extracts in terms of the antioxidant analyses. This study establishes that greenly produced ZnONPs have the potential to be used in nanomedicine to treat bacteria that are resistant to a variety of drugs, as well as those with reactive oxygen species toxicity.

## 1. Introduction

Since the beginning of time, humankind has relied on plants to meet their basic needs. The main uses of plants are food and medicine. Botanic or herbal pharmaceuticals made from plants are thought to be more affordable, less harmful, and effective in treating a variety of disorders. Multi-drug resistance (MDR) strains of bacteria are becoming increasingly difficult to treat or minimize due to evolutionary trends and resistance built into microorganism genomes. According to recent literature, the increased resistance that different microorganisms have developed to various medications, including antibiotics, is posing a serious threat to world health [1]. The current heightened resistance that microorganisms used against many multiple drugs are displaying to a variety of antibiotics is becoming a critical global health concern. The current situation makes it more important than ever to create innovative antibiotics and antimicrobial medications [1]. One method uses botanicals, which are bioactive substances derived from plants. Researchers from all over the world have documented and continue to document the roles of botanicals as antimicrobial drugs due to their effects on microbes, such as ATP loss, protein synthesis inhibition, pH disturbance, intracytoplasmic damage, and DNA damage [2]. A novel addition to these phytomedicines are nanomedicines consisting of nanomaterials, often derived from the medicinal plants, which are proving to be promising medications in biomedical research [3].

Free radicals in the body create oxidative stress, which is thought to be the primary factor in a number of diseases and conditions, including some types of tumors, ageing, and inflammatory conditions such as glomerulonephritis, vasculitis, and arthritis [4]. The body produces free radicals as a by-product of ordinary human metabolism. They can also develop as a result of exposure to environmental toxins including tobacco smoke and industrial air pollutants. In order to promote free-radical-scavenging activity and avoid cellular damage, the human body needs antioxidants to provide electrons to free radicals. In addition to being edible, cultural medicinal plants are a significant source of antioxidants, which helps them to prevent a number of ailments [5]. Therefore, there is certain need to develop promising antioxidant medications.

The environmentally friendly and cost-effective green synthesis method uses plant extracts to create nanoparticles. The synthesis fluid contains certain phytochemicals and botanicals that may improve the biocompatibility and immunomodulatory abilities of the nanoparticles. Additionally, the stability provided by the green manufacturing technique enhances the nanoparticles’ medicinal capabilities [6]. Nanotechnology is progressing quickly in the present day due to its numerous applications in biomedical research and agriculture [7]. Zinc oxide nanoparticles (ZnONPs), in particular, have applications in agriculture, sunscreens, the cosmetics industry, and food packaging [8]. Due to their capacity to penetrate the cell envelops of microbes, ZnONPs have also been found to possess antibacterial capabilities [9]. The green synthesis of nanomaterials is a potentially environmentally friendly and cost-effective way to mitigate environmental pollution. Several studies have reported the green synthesis of nanomaterials. For instance, one study [10] focused on transforming industrial and organic waste into a composite material that can effectively remove organic pollutants. The researchers synthesized the composite material by incorporating titanium-doped activated carbon and cellulose nanofibers into a paper matrix. They characterized the material using various techniques, such as SEM, XRD, and BET, and evaluated its performance in removing organic pollutants from water. Another study [11] explored the possibility of using waste materials for the green synthesis of TiO_2_ nanoparticles as a photocatalytic additive for indoor lime-based finishings. The researchers synthesized the nanoparticles using a sol–gel method with waste materials and evaluated their photocatalytic activity by incorporating them into lime-based finishings and testing their methylene blue degradation capability.

Today, there exists approximately 4500 species of angiosperm parasitic plants, yet they only account for 1% of all flowering plants [12]. *Viscum album* L. belongs to the parasitic plant family Santalaceae. In Pakistan, the usage of *V. album* for treating conditions including nausea, vomiting, cancer, diarrhea, diabetes, and hypertension is significant. It is also regarded as a crucial component of many herbal preparations, and their popularity is continuously rising [13]. Additionally, it is frequently utilized as holiday décor. It is believed to possess magical properties and is beneficial for treating a variety of urinogenital issues, fractured limbs, skin conditions, and chest conditions [14].

*V. album* is a widely distributed plant species in the AJK area, and its abundance and accessibility make it a convenient and cost-effective source for obtaining leaf extracts for nanoparticle synthesis. Furthermore, green synthesis aims to minimize the use of hazardous chemicals and energy-intensive processes. *V. album* leaf extracts offer a sustainable and environmentally friendly alternative to traditional chemical methods for synthesizing ZnONPs. Additionally, *V. album* is known to contain a rich diversity of bioactive compounds. such as phenolic compounds, flavonoids, alkaloids, and polysaccharides. These compounds possess various therapeutic properties, including antibacterial and antioxidant activities. By using *V. album* leaf extracts, a wide range of these bioactive compounds can be incorporated into the green synthesis of ZnONPs, potentially enhancing their therapeutic potential. Moreover, the combination of different bioactive compounds present in the extracts may exhibit enhanced antibacterial and antioxidant activities compared to individual compounds. Utilizing the whole extract in the synthesis process allows for the preservation of these synergistic effects, potentially resulting in nanoparticles with improved biological properties.

Little work has been reported in the literature on the use of *V. album* in the green synthesis of ZnONPs, but the literature reports a few studies utilizing *V. album* for a green synthesis approach. For instance [15], researchers used *V. album* leaf extract for the green synthesis of ZnONPs. They characterized the nanoparticles using various techniques, such as UV–Vis spectroscopy, XRD, SEM, and TEM. They also evaluated the antimicrobial activity of the synthesized nanoparticles against various bacterial strains. The study found that the *V. album* leaf extract was an effective reducing and capping agent for the synthesis of ZnONPs. The synthesized nanoparticles were spherical and had a mean particle size of around 20 nm. The antimicrobial activity assessment showed that the ZnONPs had a significant inhibitory effect on the growth of the tested bacterial strains. In another study [16], the researchers used *V. album* extract as a reducing and capping agent to synthesize ZnONPs. They characterized the nanoparticles using various techniques and evaluated the cytotoxicity of the nanoparticles using the MTT assay on the MCF-7 breast cancer cell line. The study concluded that *V. album* extract was an effective green reducing agent for the synthesis of ZnONPs. The synthesized nanoparticles were found to be crystalline and had a hexagonal wurtzite structure. The cytotoxicity assessment showed that the ZnONPs had a dose-dependent cytotoxic effect on the MCF-7 cell line. The above studies provide convincing evidence that plant extracts of *V. album* might be used in the green synthesis of ZnONPs.

The current work is based on the GCMS analysis of some volatile compounds found in the *V. album* leaf extracts. The aim of the study was to investigate and compare the chemical compositions and antibacterial and antioxidant properties of *V. album* leaf extracts with green-synthesized zinc oxide nanoparticles. Furthermore, the study aimed to assess the potential of green-synthesized zinc oxide nanoparticles as novel antimicrobial and antioxidant agents. The objectives of the study included an analysis of the chemical composition of *V. album* leaf extracts using GCMS, aiming to identify the major compounds present in the extracts and to determine the antibacterial activity of *V. album* leaf extracts and green-synthesized zinc oxide nanoparticles against a range of pathogenic bacteria. Furthermore, the antioxidant activity of *V. album* leaf extracts and green-synthesized zinc oxide nanoparticles was evaluated using in vitro assays, and the antibacterial and antioxidant activities of *V. album* leaf extracts with green-synthesized zinc oxide nanoparticles were compared. The authors seek to provide an insight into the potential use of *V. album* leaf extracts and green-synthesized zinc oxide nanoparticles in the development of new therapeutic agents for the treatment of infectious diseases and oxidative-stress-related disorders. The leaf extracts of *V. album* might serve as a promising medium for green nano-zinc oxide synthesis. The roles of ZnONPs and methanolic leaf extracts of *V. album* are compared for their antimicrobial efficacy and antioxidant activities. We believe that green-synthesized ZnONPs might serve as better antimicrobial agents and might be useful in addressing the challenge of antimicrobial resistance (AMR). The effectiveness of the plant as a potential source of green medicines against microbes is shown through this investigation. The comprehensive research aim is to understand the green synthesis of ZnONPs using *V. album* extracts and explore their antibacterial and antioxidant properties. This study aims to contribute to the development of sustainable and eco-friendly nanomaterials with potential biomedical applications.

## 2. Results

### 2.1. GC-MS Analysis of Methanolic Extracts

The identification was performed by comparing the linear retention index to the hydrocarbons and available MS literature. A detailed summary of the compounds identified is presented in Table 1.

### 2.2. Total Phenolics and Free Proline Contents

The assayed total phenolics and leaf free proline contents are presented in Table 2. The presence of phenolics and free proline contents in both aqueous and methanolic extracts varied slightly. The proline contents were assayed to demonstrate that the source plant was not under stress. Usually, elevated levels of free proline indicate that a plant is exposed to drought stress or some oxidative damage. Our analysis shows that the plant is a rich source of botanicals.

### 2.3. Green Synthesis and Characterization

**Figure 1:**
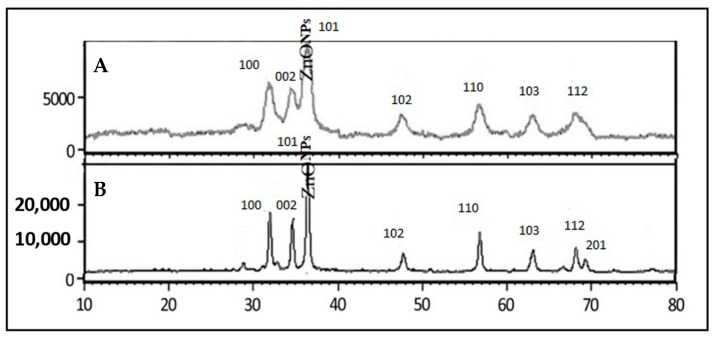
XRD patterns of ZnONPs. (**A**) XRD pattern of ZnONPs synthesized using methanolic extracts. (**B**) XRD pattern of ZnONPs synthesized using aqueous extracts. XRD patterns were recorded in a range from 10° to 80° 2θ.

The XRD patterns of ZnONPs synthesized from different origins, such as different solvent extracts, differ due to differences in the synthesis conditions and resulting particle sizes and crystal structures. As shown in Figure 1A, the methanolic extracts produced ZnONPs with smaller particle sizes, which resulted in broader diffraction peaks and changes in the peak positions and intensities compared to those obtained from larger particles synthesized using aqueous extracts, as shown in Figure 1B. The (100), (002), (101), (102), (110), (103), (112), and (201) reflection lines of the ZnONPs were identified at 2θ angles of 31.83, 34.48, 36.32, 47.60, 56.66, 62.97, 68.01, and 69.09 based on the peak positions observed in the XRD patterns. The Debye–Scherrer equation was employed to determine the size of the nanoparticles, as mentioned below.

D = K × λ/β × cos(θ)

Above, D refers to the average crystallite size of the nanoparticles. It represents the characteristic dimension of the nanoparticles being studied. K represents a dimensionless shape factor, which depends on the geometry of the sample and the X-ray diffraction setup. It is typically a constant specific to the experimental setup (in our case, we used 0.9 as an approximation). Λ denotes the wavelength of the X-ray used in the experiment (0.1542 nm). It is usually expressed in units of length, such as meters or angstroms. Β represents the full width at half maximum (FWHM) of the diffraction peak in the X-ray diffraction pattern. It is a measure of the broadening of the peaks due to various factors, such as the size and strain of the crystalline domains. θ denotes the Bragg angle, which is the angle between the incident X-ray beam and the crystal lattice planes in the sample. It is typically measured in degrees. By plugging in the appropriate values for these variables, the Debye–Scherrer equation allows for the determination of the average crystallite size of the nanoparticles based on their X-ray diffraction patterns. The average crystalline size of the formed nanoparticles was estimated using Scherrer’s formula, resulting in values of 39.82 nm and 8.14 nm for the ZnONPs green-synthesized using aqueous and methanolic extracts of *V. album*, respectively.

**Figure 2:**
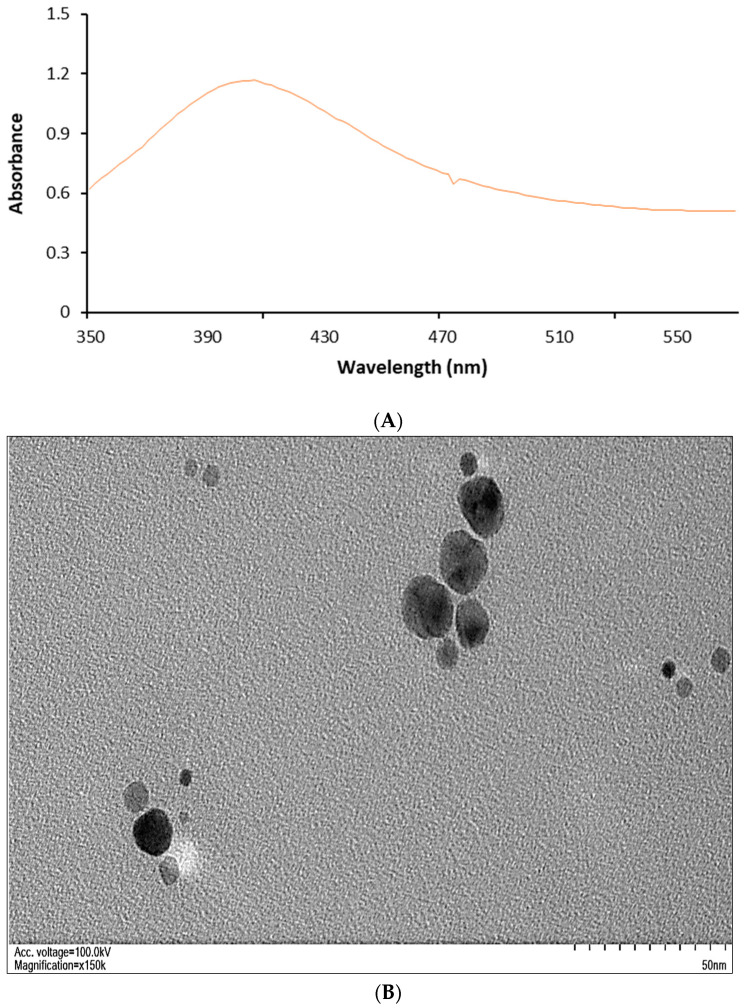

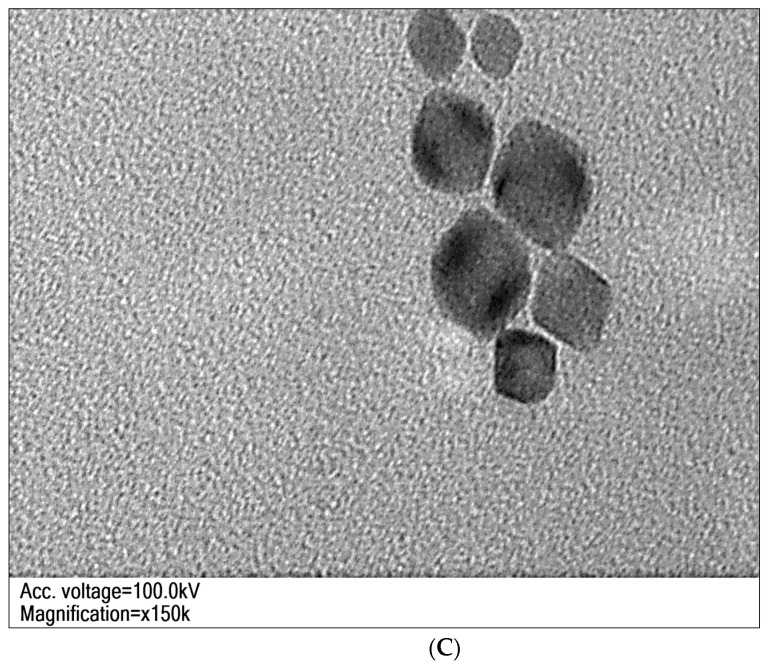
(**A**). Absorbance spectra of ZnONPs green-synthesized from the methanolic extracts of *V. album* peaking at 406 nm. (**B**). TEM image of ZnONPs synthesized from methanolic extract showing a quasi-spherical to spherical shape and size distribution averaging 13.5 nm. (**C**). TEM micrograph of the ZnONPs green-synthesized using aqueous extracts of *V. album*. The particles were larger in size and had smaller surface areas.

The methanolic-extract-mediated ZnONPs’ UV–visible peak was noted at 406 nm, indicating that the particles were synthesized (Figure 2A). TEM pictures of these ZnONPs are shown in Figure 2B. A copper grid with a distinctive carbon layer was employed for imaging purposes. The 98.7% pure ZnONPs green-synthesized using methanolic extracts of *V. album* employed in the experimental trial had a surface area of 178 m^2^·g^−1^ (BET Surface Area and Porosity Analyser, Model: Tristar II 3020, manufactured by Micromeritics Instrument Corporation based in Norcross, GA, USA) and a 5.1 Kg·L^−1^ density and were green in color. The ZnONPs with quasi-spherical forms are shown to have an average size distribution of 13.5 nm in the TEM images (Figure 2B).

The TEM micrographs for the ZnONPs synthesized using aqueous plant extract are shown in Figure 2C. These particles were quasi-spherical, having a larger particle size averaging 50 nm with a smaller surface area of around 13 m^2^/g.

### 2.4. Antibacterial Activities

The antimicrobial efficacy of nano-zinc oxide derived from *V. album* and the source plant extracts, in terms of the zone of inhibition, is shown in Table 3 and was compared to that of the standard drug, moxifloxacin (25 μg/mL). The maximum zone of inhibition was recorded for the ZnONPs prepared from aqueous extracts of *V. album.* The green-synthesized ZnONPs showed better antibacterial behavior compared to the source plant extract.

### 2.5. DNA Gyrase-B Inhibition

**Figure 3:**
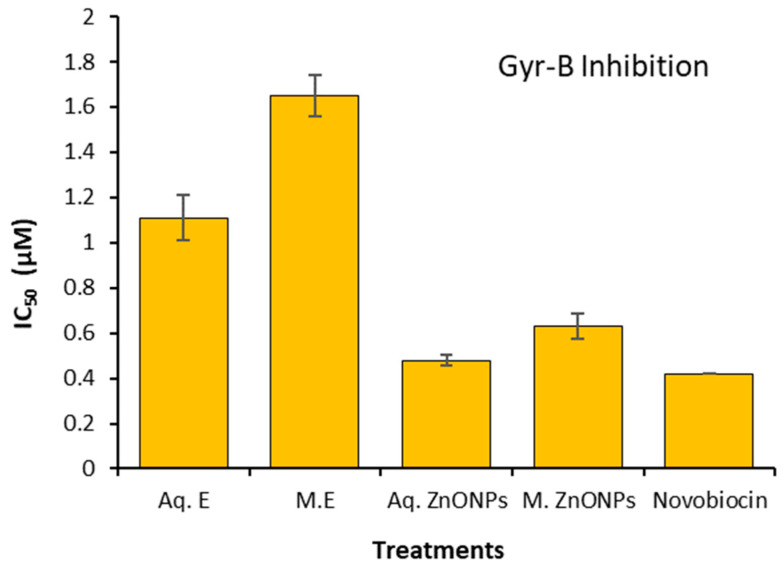
Invitro Gyr-B activity as demonstrated through the application of test extracts from *V. album* and green-synthesized ZnONPs. Aq: aqueous; E: extracts; M: methanolic; ZnONPs: zinc oxide nanoparticles (Mean ± S.E; n = 3).

Observations were recorded regarding the invitro DNA Gyrase-B (Gyr-B) inhibition for the test extracts of the green-synthesized ZnONPs and source plant *V. album.* The results were compared to the standard drug, novobiocin (Figure 3). The results show that green-synthesized ZnONPs are potent inhibitors of Gyr-B activity, and their effects are comparable to the effects produced by novobiocin. Aqueous extracts of *V. album* were found to be least effective in limiting Gyr-B activity.

### 2.6. Antioxidant Potential

**Figure 4:**
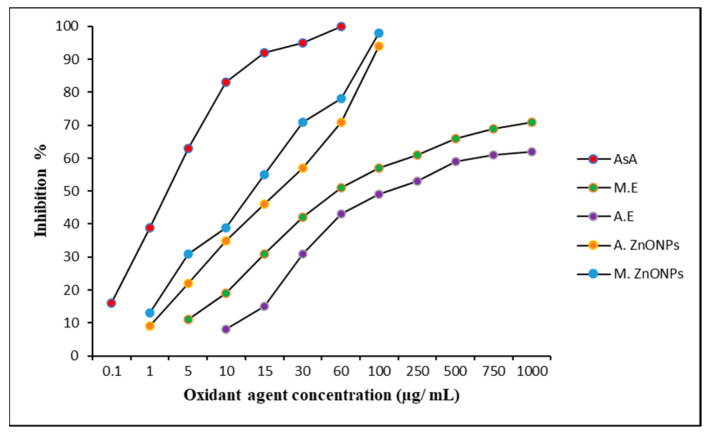
Antioxidant potential of green-synthesized ZnONPs and source plant extracts. As A: ascorbic acid; M.E.: methanolic extract of *V. album*; A.E.: aqueous extract of *V. album*; A. ZnONPs: zinc oxide nanoparticles green-synthesized using aqueous extracts of *V. album*; and M. ZnONPs: ZnONPs green-synthesized using methanolic extracts of *V. album*.

Figure 4 provides an illustration of the antioxidant activity of the green-produced ZNONPs and *V. album* leaf extracts. Compared to aqueous extracts, methanolic extract has stronger antioxidant activity. According to the study’s findings, at a dosage of 750 μg/mL, the plant extracts can scavenge 80% of DPPH radicals, whereas ascorbic acid, a common antioxidant, can do so at a concentration of 15.0 μg/mL. The effects of the methanolic ZnONPs were the best of all the test extracts, and they were able to demonstrate promising antioxidant potential. The aqueous- and methanolic-extract-mediated green ZnONPs, at a concentration of 100 μg/mL, were able scavenge 94% and 98% of DPPH free radicals.

## 3. Materials and Methods

### 3.1. Plant Collection and Extraction

The local peasant association of Mirpur Azad Jammu and Kashmir (AJK), Pakistan, provided the tree species of Quercus dilatata from which the *V. album* plants were harvested. The sampling process was finished in November 2021. A sonicator produced by ISO LAB Germany was used to sonicate 40 grammes of the crushed herb in 1 L of methanol for 30 min at 40 °C, after which it was kept for 24 h in the dark. Using medium-grade filter paper, the mixture was purified, and methanol was added to bring the level to 1 L. The same procedure was used to create the aqueous extracts, which required 1 L of distilled water [17].

### 3.2. GC-MS Analysis of the Methanolic Extracts

A Shimadzu QP2020 GC-MS (Shimadzu Corporation, Kyoto, Japan) equipped with a split–splitless-type injector was used to analyze the methanol extracts following the method described by Naik et al. [18]. The mass spectrum of each original component was examined and compared to the literature data from the libraries of NIST 2017 and ADAMS-2007.

### 3.3. Determination of the Total Phenolic and Proline Contents of Extracts from the Sample Plant

The technique described by [19] was used to determine the total free proline concentration. Using the recommended reagents, frozen leaves were extracted through a process that involved centrifugation, boiling, and cooling. Readings were taken at 520 nm, and toluene was used as the reference. Using a calibration curve, the amount of proline was calculated and expressed as mg proline g^−1^ FW.

Total phenolics were assayed following [20]. Table 4 outlines the reagent values used in the method.

### 3.4. Green Synthesis and Characterization of Zinc Oxide Nanoparticles

The use of either methanolic or aqueous extracts of *V. album* may have an impact on the size and shape of the synthesized ZnONPs; thus, the choice of extract was considered in relation to the intended application of the nanoparticles. Additionally, to ensure reproducibility, the experimental conditions (such as pH, temperature, and stirring speed) were kept consistent between different batches of synthesized ZnONPs. The choice of solvent, whether aqueous or methanolic extracts of *V. album* are investigated, can affect the size and shape of the synthesized ZnONPs. This is because the solvent used can affect the reaction kinetics and, thus, the nucleation and growth rate of the nanoparticles.

A liter of 0.01 M zinc acetate dihydrate solution was prepared in distilled water. Using a magnetic stirrer, the zinc acetate dihydrate solution was vigorously agitated as 25 mL of herbal extract (either methanolic or aqueous extract) was added dropwise. The mixtures were heated to 70 °C for 30 min under constant stirring, and the pH of the reaction mixture was adjusted to 9 using sodium hydroxide (NaOH) for the purpose of the experiment. The combination abruptly changed color to a deep black hue. Subsequently, the resulting suspension was centrifuged for 15 min at 10,000 rpm using a Hermle Z326 centrifuge (Labtechnik GmbH, Wehingen, Germany), and the synthesized ZnONPs were washed three times with deionized water to remove any unreacted reagents or impurities. The NPs were collected and dried for further analysis [18].

Utilizing transmission electron microscopy and UV–visible spectroscopy, the characterization of NPs was carried out at the Department of Science, University of Central Punjab (UCP), Lahore, Punjab, Pakistan. Using an ELICO SL-159 Spectrophotometer (Suzhou, China) in the 350–570 nm region, a UV–Vis spectral analysis was performed. These samples’ transmission electron microscopy (TEM) pictures were captured using an FEI Tecnai 12 from Gatan, Germering, Germany that was accessible at the Institute of Space Technology (IST), Islamabad [21,22]. The crystal structure of the nanoparticles was investigated through powder X-ray diffraction (XRD) using a Phillips X Pert Model X-ray diffractometer, PANalytical, Almelo, Netherlands. CuKα radiation with a wavelength of 0.1542 nm was used, and the XRD pattern was recorded in a range from 10° to 80° 2θ.

### 3.5. Antibacterial Activities

The test bacterial strains included Pseudomonas aeruginosa (ATCC 10145), Escherichia coli (ATCC 10799), Staphylococcus aureus (ATCC 29213), and Bacillus subtilis (ATCC 11774). We determined whether the extract, silver nanoparticles, and the common antibiotic moxifloxacin could all limit bacterial growth. The inhibition zones produced by the inoculum of the studied bacteria were measured on 9 cm plates filled with sterile nutrient agar. Using a 6 mm puncher that had been sanitized, a 6 mm well was created. Then, 40 L of test solution (25 g/mL) and the moxifloxacin standard solution was poured into the wells. The control solution was 5% DMSO. At 37 C, the plates were incubated for 24 h. The outcomes of every experiment were replicated [23]. Gyr-B activity was assayed following [24].

### 3.6. Determination of Antioxidant Potential

The strategy described in [25] was applied with a few changes. In brief, 0.15 mL of 0.2 mM DPPH solution (dissolved in MeOH) was added to 0.15 mL of each extract solution. A Synergy HTC multimode reader was used to assess the solutions’ absorbance at 517 nm in comparison to a methanol blank after 30 min of dark incubation at room temperature. The ability to scavenge the DPPH radical was calculated using the equation below:

DPPH Scavenging activity (%) = (Absorbance of blank-absorbance of extract)/(absorbance of blank) × 100.

## 4. Discussion

The hemi-parasitic herb *V. album* has been identified as a traditional ethnomedicine in several rural and hilly areas of AJK, Pakistan. Ethnobotanical and folklore research offered the original, fundamental foundation for selecting this plant for further phytochemical and analytical exploration or the isolation of single components with some efficacy and reactivity for managing different bacterial diseases and strong antioxidant potential. The methanolic extract of *V. album* was analyzed using GC-MS, revealing the presence of nonadecane and 2-methyltricosane, which are naturally occurring compounds in *Astragalus mongholicus* and *Achillea millefolium* [26]. These compounds have been identified as natural pest killers and growth regulators in plants. Additionally, the presence of potential antioxidants, di-tert-butyl-4-methylphenol and 2,4-di-tert-butylphenol, was detected in *V. album* [27]. Octacosane, a hydrocarbon compound with 28 carbon atoms, was identified as a natural product in Mikania cordifolia and is found in various plant species [28]. GC-MS analysis also revealed the presence of n-tetradecane, which is commonly used in organic synthesis. The methanolic extracts showed the presence of liquid alkanes, including 2-methylundecane. Tridecane, a natural product of *Juglan nigra*, was also noted [29]. Heneicosane, on its own, is primarily considered an inert hydrocarbon with minimal biological activity. However, it is important to note that heneicosane can contribute to the physical properties of substances or organisms in which it is found, for example, by acting as a component of the cuticle in plants and providing protection against moisture loss and physical damage. The presence of eicosane, a major antifungal metabolite, was observed in the extracts, and it exhibits antimicrobial properties. Furthermore, the methanolic extracts of *V. album* were found to contain heneicosane [30], which is reported as a potential antimicrobial alkane produced by plants. Additionally, the GCMS of the extract revealed the presence of pentadecane and heptadecane, which are major hydrocarbons and have been reported for their antimicrobial activity [31]. The compound is also a potential antigingivitic. The compounds 6-phenyldodecane and 5-phenyldodecane have been reported in *Abrus precatorius*, with no potential activity identified thus far [32]. The results of GC-MS are in accordance with the work reported in [33]. In the GC-MS analyses, a total ion chromatogram (TIC) was produced, which is a plot of the sum of the intensities for all ions in a predetermined range as a function of time. It is often helpful to take the average of the mass spectra gathered across a peak to identify an unknown analyte. To enhance chemical identification with library searching, background ions can also be removed. Furthermore, for the current study, methanolic extracts of *V. album* were preferred, since methanol has a high solubility power, which makes it a good choice for extracting a wide range of compounds from plants, including bioactive molecules with antimicrobial properties. Methanol is a non-polar solvent, which means that it can extract a broad range of compounds, including lipids and non-polar secondary metabolites that are often associated with antimicrobial activity. Moreover, methanol is a relatively stable solvent and is less prone to oxidation or degradation than other solvents, making it an ideal solvent for the long-term storage of plant extracts. Additionally, methanol is a safe and inexpensive solvent compared to other solvents such as chloroform, acetone, or ethanol, which are toxic or flammable [34].

Plants store proline and soluble carbohydrates to regulate their osmotic potential. The proline content of leaves is understood to be one of the earliest indicators of stress [35]. Variables including the kind of stress and length of exposure may play a role in the low proline concentration of *V. album* leaves. The proline content of the leaves suggests that under the conditions used in this study, stress was not a factor for *V. album*. Proline catabolism may be successfully stimulated in leaves that are in the last stages of senescence, during drought stress, and during changes in nutritional status [36]. According to our findings, *V. album* is a rich source of biocomponents, and its extracts, when grown under typical conditions, have the ability to reduce zinc ions. Overall, the finding of this research support the contribution of phenolics to this plant’s antioxidant and anti-inflammatory properties [37].

In the current study, we used a green synthesis method to create ZnONPs. It has been demonstrated that between 330 and 460 nm, zinc oxide nanoparticles exhibit a characteristic broad absorption peak [38]. The production of zinc nanoparticles from *V. album* leaf extracts is thus successfully demonstrated by an optical absorption band peaking at 406 nm. This quick extracellular precipitation process has the potential to become a straightforward method for producing nanoparticles [39]. To understand the crystalline characteristics and size of the nanoparticles, a TEM study was carried out. The particles were nearly quasi-spherical in form, with only a slight thickness fluctuation, as shown in the ZnO TEM images. This research supports the findings of [22,39,40]. The methanolic extracts produced ZnONPs with smaller particle sizes, which resulted in broader diffraction peaks and changes in the peak positions and intensities compared to those obtained from larger particles synthesized using aqueous extracts. When a material is analyzed with XRD, a beam of X-rays is directed towards the sample, and the resulting diffracted X-rays are detected and measured. The XRD pattern produced by a material is a plot of the intensity of the diffracted X-rays versus the angle of diffraction. The width of the diffraction peak reflects the crystalline size of the material, with broader peaks indicating smaller crystallites. When a material has a larger surface area, this often means that it has smaller crystallites, which can result in broader XRD peaks. This is because smaller crystallites have fewer atoms in the crystal lattice, which results in a wider distribution of scattering angles and broader peaks in the XRD pattern. Additionally, it was noticed that smaller particles, produced via methanolic-extract-mediated green synthesis, had a larger surface area compared to the-aqueous extract-mediated green-synthesized ZnONPs, which had a smaller surface area. This smaller particle size and higher surface area of ZnONPs produced using methanolic extracts might be due to the higher solubility of Zn precursors in organic solvents. The above findings are in good agreement with the pool of previous studies [41,42].

The measured particle sizes of the ZnONPs differed for both TEM and XRD. Significant discrepancies in particle sizes, as measured through TEM and XRD, are not uncommon and can be attributed to various factors. TEM provides a direct visualization of individual particles, offering a more precise measurement of particle size by capturing the actual morphology and dimensions. On the other hand, XRD calculates the average crystallite size based on diffraction patterns, which can be influenced by factors such as crystallite shape, strain, and preferred orientation. The measurement principles of these techniques differ, leading to variations in the reported sizes. Additionally, the sample preparation techniques for TEM and XRD can introduce artifacts and affect the size measurements due to factors such as aggregation, drying effects, or uneven distribution. The limitations and calibration of the instruments used, as well as the heterogeneity of the particle samples themselves, can also contribute to the observed discrepancies [41,42].

The green production method is currently preferred for reducing the harmful problems and dangerous effects of nanoparticles. The green synthesis method ensures the controlled and targeted distribution of nanomedicine, and it is supported by the scientific community at large. Additionally, there are fewer adverse effects associated with the development of nanoparticles using a green synthesis method [43]. The controlled release of the nanomedicine is made possible by the plants’ bioactive substances and natural botanicals [44]. According to the proposed mechanism, combinations of compounds, as extracts, were utilized in the green synthesis of ZnONPs. Based upon the previous literature [15,16], we used a combination of extracts in green synthesis. The use of plant extracts as reducing agents, stabilizers, or capping agents for nanoparticle formation is crucial in the green synthesis approach.

The behavior of the green-produced ZnONPs, in terms of their antibacterial properties, was improved compared to the source plant extracts. These results could be attributed to the green synthesis’ non-hazardous and safe methodology, which uses plant extracts as capping and reducing agents [45]. The synthesized ZnONPs may not necessarily contain the exact same bioactive compounds as those found in the extracts. The green synthesis process may selectively incorporate certain compounds from the extracts or lead to the formation of new compounds during the nanoparticle synthesis. This might be a possible reason for the better antibacterial activities of ZnONPs compared to source plant extracts. The antibacterial properties of ZnONPs were further enhanced through the use of aqueous extracts in green synthesis. This can be explained by the greater negative charge and more homogeneous size distribution of aqueous extract nanoparticles compared to methanolic extract nanoparticles. The synthesized ZnONPs are expected to exhibit higher stability compared to the extracts, which may degrade over time due to various factors, such as exposure to light, heat, or enzymatic degradation. Thus, presumably, the better antibacterial properties of green-synthesized ZnONPs, as compared to plant extracts, are due to their stability. Several researchers have reported on the function of *V. album* leaf extracts as antibacterial agents [46,47]. The ZnONPs are known to interfere with bacterial DNA replication and drastically change how virulence-causing genes are expressed in the bacteria, which limits the pathogenic potential of the bacteria [48]. In the green synthesis method, phenolics and flavonoids found in plants serve as the primary reducing and capping agents. By blocking the activity of DNA gyrase, these flavonoids have been found to induce the negative supercoiling of bacterial DNA [49]. Gyr-B structural dynamics indicate that the enzyme is composed of two subunits (alpha and beta subunits). Antibiotics target the alpha subunit to interfere with bacterial DNA amplification. According to Singh et al. [50], the application of ZnONPs causes an upregulation of genes such as those for gyrase and single-stranded binding protein, which prevents bacteria from replicating their DNA and encourages bacterial mortality and stagnant development. These results provide proof of our current findings. Additionally, according to Slade and Radman [51], the application of ZnONPs causes a downregulation of genes involved in DNA repair mechanisms, such as base excision and mismatch repair.

The antioxidant potential of the ZnONPs was higher compared to the source plant extract, and the methanolic extracts were better at enhancing antioxidant potential. For Tunisian brachychiton, the methanolic extract had better antioxidant activity than the aqueous extract of the leaves [52]. The existence of alpha-tocopherol and other undiscovered phytochemicals in plant extracts, as well as the presence of phenolics and flavonoids, may contribute to the antioxidant action. Additionally, the synergistic effects of the extract’s numerous biocomponents, each of which has a unique structure, polarity, and capacity to dissolve in a particular solvent, will affect the antioxidant capacity [53,54].

According to our results, ZnONPs proved effective on most occasions, as compared to the source plant extracts. ZnONPs have gained significant attention in antibacterial and antioxidant studies due to their unique properties and superior performance compared to plant extracts. ZnONPs possess a large surface-area-to-volume ratio, which allows for a larger contact area with bacterial cells or reactive oxygen species (ROS) in antioxidant studies. This increased contact enhances the interaction between the nanoparticles and the target, leading to enhanced antibacterial and antioxidant effects [16,21]. In contrast, the source plant extracts contained a mixture of compounds, as verified by the GCMS study, and their activity may vary depending on the extraction method and plant source, making them less reliable and consistent. ZnONPs exhibit potent antibacterial properties by inducing oxidative stress in bacteria. They generate reactive oxygen species (ROS) such as hydrogen peroxide (H_2_O_2_), superoxide ions (O_2_), and hydroxyl radicals (OH-) that can damage bacterial cell membranes, proteins, and DNA, leading to bacterial death. In comparison, *V. album* extracts may contain different active compounds that can have varying degrees of antibacterial activity, and their mechanisms of action can be more complex and less well-understood. Additionally, ZnONPs demonstrate long-lasting stability and the sustained release of antibacterial and antioxidant agents, which can provide extended protection against bacteria and oxidative damage. Their stable structure allows them to retain their activity over time, whereas plant extracts can degrade or lose their potency due to environmental factors, storage conditions, or interactions with other components [9,16,21].

## 5. Conclusions

A GC-MS analysis of *V. album* methanolic extracts was conducted. The analysis showed that the plant is enriched with botanicals and volatile compounds useful for various biomedical applications. The green synthesis of nano-zinc oxide was accomplished using both the aqueous extracts and methanolic extracts of *V. album*, and the method was confirmed in its success through characterization techniques. The XR diffractograms revealed that methanolic extracts were associated with smaller particle sizes, which resulted in broader diffraction peaks and changes in the peak positions and intensities as compared to those obtained from larger particles synthesized using aqueous extracts. A comparative study on the antimicrobial efficacy and antioxidant potential of the synthesized ZnONPs and their source extracts was conducted, which revealed that the green-synthesized ZnONPs might be a potential nanomedicine for treating microbial infections. The aqueous extracts of the ZnONPs were found to more reliably limit bacterial growth. Both aqueous- and methanolic-extract-mediated green ZnONPs showed significant antioxidant activity, with methanolic extracts showing better efficacy. From the perspective of antimicrobial resistance, we recommend the use of green-synthesized nanoparticles as substitutes for antibiotic drugs.

## Figures and Tables

**Table 1:** Phytochemical constituents of *Viscum album* plant methanolic extract derived via GC-MS.

S.NO.	Systematic Name	Molecular Formula	CAS #	Mol. Mass g/mol	Retention Time Min.	Calculated Retention Indices	Documented Retention Indices
1	*n*-Tetradecane	C_14_H_30_	629-59	198.3	4.940	837	239.35
2	*n*-Decane	CH_3_(CH_2_)_8_CH_3_	124-18-5	142.2	4.940	837	163.22
3	2-Methylundecane	C_12_H_26_	7045-71-8	170.3	4.940	837	1165.1
4	5-Ethyl-2-methyloctane	C_11_H_24_	62016-18-6	156.31	5.368	858	-
5	*n*-Hexadecane	C_16_ H_34_	544-76-3	226.4	5.368	858	272.02
6	2,3-Dimethylundecane	C_13_H_28_	17312-77-5	184.36	5.368	858	-
7	*n*-Dodecane	C_12_H_26_	112-40-3	170.3	6.839	927	202.83
8	*n*-Tridecane	CH_3_(CH_2_)_11_CH_3_	629-50-5	184.37	6.839	927	221.55
9	2,6-Dimethylundecane	C_13_H_28_	17301-23-4	184.36	7.069	937	1210
10	Benzene, 1,3-bis(1,1-dimethylethyl)-	C_14_H_22_	1014-60-4	190.32	7.920	974	-
11	*n*-Pentadecane	C_15_H_32_	629-62-9	212.41	8.481	999	256.12
12	*n*-Eicosane	C_20_H_42_	112-95-8	282.5	8.481	999	333.60
13	2-Methyloctane	C_9_H_20_	3221-61-2	128.2	9.743	1054	864.90
14	1-Iodo-2-methylnonane	C_10_H_21_I	101-47-9	268.17	9.743	1054	
15	*n*-Heptadecane	C_17_H_36_	629-78-7	240.47	11.557	1135	282.99
16	*n*-Nonadecane	C_19_H_40_	629-92-5	268.52	11.851	1148	
17	2-Methyltricosane	C_24_H_50_	1928-30-9	338.66	11.851	1148	2365
18	1-Iodotetradecane	C_14_H_29_I	19218-94-1	324.28	11.851	1148	
19	2-Methyldodecane	C_13_H_28_	1560-97-0	184.36	12.610	1183	1261
20	2,6,10,14-Tetramethylheptadecane	C_21_H_44_	018344-37-1	296.574	12.701	1187	
21	5-Propyldecane	C_13_H_28_	17312-62-8	184.36	12.701	1187	
22	1-Iodododecane	C_12_H_25_I	4292-19-7	296.23	13.102	1206	
23	*n*-Octacosane	C_28_H_58_	630-02-4	394.77	13.177	1210	442.42
24	Methoxyacetic acid, 2-tetradecyl ester	C_17_H_34_O_3_	282-04-8	286.4	13.316	1216	
25	10-Methylnonadecane	C_20_H_42_	56862-62-5	282.55	13.316	1216	
26	*n*-Heneicosane	C_21_H_44_	629-94-7	296.58	13.349	1218	348.41
27	*n*-Tetradecane	C_14_H_30_	629-59-4	198.39	13.349	1218	239.35
28	2,6-Di-tert-butyl-4-methylphenol	C_15_H_24_O	128-37-0	220.35	13.488	1225	1514.
29	2,4-Di-tert-butylphenol	C_14_H_22_O	96-76-4	206.32	13.536	1227	1512
30	Methoxyacetic acid, 2-tridecyl ester	C_16_H_32_O_3_	282-04-5	272.42	13.664	1233	
31	Octadecane-1-sulfonyl chloride	C_18_H_37_ClO_2_S	342-70-4	353.0	13.664	1233	
32	Benzene, 1-propylheptyl	C_16_H_26_	4537-12-6	218.38	14.162	1257	
33	Benzene, 1-ethyloctyl	C_16_H_26_	4621-36-7	218.37	14.509	1274	
34	Benzene, 1-pentylhexyl	C_17_H_28_	4537-14-8	232.4	15.632	1330	
35	Benzene, 1-butylheptyl	C_17_H_28_	4537-15-9	232.4	15.691	1333	
36	Benzene, 1-propyloctyl	C_17_H_28_	4536-86-1	232.4	15.878	1343	
37	Tetradecane, 4-ethyl	C_16_H_34_	55045-14-2	226.44	16.750	1388	
38	Dodecane, 2-methyl-6-propyl	C_16_H_34_	55045-08-4	226.44	16.750	1388	
39	Docosane, 11-butyl	C_26_H_54_	013475-76-8	366.71	16.814	1391	
40	Undecane, 2-phenyl	C_17_H_28_	4536-88-3	232.41	16.911	1396	
41	Dodecane, 6-phenyl	C_18_H_30_	2719-62-2	246.4	17.290	1417	
42	Dodecane, 5-phenyl	C_18_H_30_	2719-63-3	246.4	17.371	1421	
43	Tetradecane, 2-bromo	C_14_H_29_Br	74036-95-6	277.28	17.510	1429	
44	Benzene, 1-ethyldecyl	C_18_H_30_	2400-00-2	246.4	17.964	1454	

**Table 2:** Free proline and total phenolics assayed in the leaves of *V. album*.

Sample	Leaf Free Proline (mg·g^−1^·FW)	Total Phenolics (mg GAE/g Extract)
Aqueous extracts of *V. album*	10.23 ± 0.12	6.43 ± 0.56
Methanolic extracts of *V. album*	9.28 ± 0.11	8.22 ± 0.43

**Table 3:** Zone of inhibition under the influence of ZnONPs and source plant extracts of *V. album*.

Test Medicine	*E. coli*		*S. aureus*		*P. aeruginosa*		*B. subtilis*	
	Aq.	Met.	Aq.	Met.	Aq.	Met.	Aq.	Met.
Source plant extract of *V. album*	36 ± 0.9	34 ± 0.4	24 ± 0.8	23 ± 0.8	25 ± 0.2	22 ± 0.2	22 ± 0.9	21 ± 0.3
Gree-synthesized ZnONPs	44 ± 0.2	43 ± 0.2	40 ± 0.3	39 ± 0.3	36 ± 0.9	33 ± 0.6	21 ± 0.7	20 ± 0.7
Standard drug	45 ± 0.4		41 ± 0.6		35 ± 0.5		22 ± 0.5	

n = 3 measurements, diameter of well = 6 mm.

**Table 4:** Composition of different solutions used for the total phenolic content (TPC) assay.

Chemicals Used	Conc. Used	Final Amount Used (500 mL)
Folin–Ciocalteu reagent	2 N	0.1 mL
Na_2_CO_3_	10%	2.8 mL
Plant Extract	0.5 mg/mL	100 μg/mL

## Data Availability

All data are available within this publication.
